# Dual inhibition of HDAC and EGFR signaling with CUDC-101 induces potent suppression of tumor growth and metastasis in anaplastic thyroid cancer

**DOI:** 10.18632/oncotarget.3268

**Published:** 2015-04-13

**Authors:** Lisa Zhang, Yaqin Zhang, Amit Mehta, Myriem Boufraqech, Sean Davis, Jing Wang, Ze Tian, Zhiya Yu, Matthew B. Boxer, Jeffrey A. Kiefer, John A. Copland, Robert C. Smallridge, Zhuyin Li, Min Shen, Electron Kebebew

**Affiliations:** ^1^ Endocrine Oncology Branch, National Cancer Institute, National Institutes of Health, Bethesda, MD, USA; ^2^ Division of Pre-Clinical Innovation, National Center for Advancing Translational Sciences, National Institutes of Health, Bethesda, MD, USA; ^3^ Geisel School of Medicine at Dartmouth, Hanover, New Hampshire, Medical School, UK; ^4^ Cancer Genetics Branch, Center for Cancer Research, National Cancer Institute, Bethesda, MD, USA; ^5^ Curis, Inc., Translational Science MA, USA; ^6^ Surgery Branch, National Cancer Institute, National Institutes of Health, Bethesda, MD, USA; ^7^ Division of Information Sciences, Translational Genomics Research Institute, Phoenix, AZ, USA; ^8^ Department of Cancer Biology, Mayo Clinic, Jacksonville, FL, USA; ^9^ Endocrinology Division, Internal Medicine Department, Mayo Clinic, Jacksonville, FL, USA

**Keywords:** CUDC-101, anaplastic thyroid cancer, quantitative high-throughput screening, EGFR, HDAC

## Abstract

Anaplastic thyroid cancer (ATC) is one of the most lethal human malignancies that currently has no effective therapy. We performed quantitative high-throughput screening (qHTS) in three ATC cell lines using 3,282 clinically approved drugs and drug candidates, and identified 100 active agents. Enrichment analysis of active compounds showed that inhibitors of EGFR and histone deacetylase (HDAC) were most active. Of these, the first-in-class dual inhibitor of EGFR, HER2 and HDACs, CUDC-101, had the highest efficacy and lower IC_50_ than established drugs. We validated that CUDC-101 inhibited cellular proliferation and resulted in cell death by inducing cell cycle arrest and caspase-dependent apoptosis. CUDC-101 also inhibited cellular migration *in vitro*. Mechanistically, CUDC-101 inhibited MAPK signaling and histone deacetylation in ATC cell lines with multiple driver mutations present in human ATC. The anticancer effect of CUDC-101 was associated with increased expression of p21 and E-cadherin, and reduced expression of survivin, XIAP, β-catenin, N-cadherin, and Vimentin. In an *in vivo* mouse model of metastatic ATC, CUDC-101 inhibited tumor growth and metastases, and significantly prolonged survival. Response to CUDC-101 treatment *in vivo* was associated with increased histone 3 acetylation and reduced survivin expression. Our findings provide a preclinical basis to evaluate CUDC-101 therapy in ATC.

## INTRODUCTION

Anaplastic thyroid cancer (ATC) is one of the most lethal human malignancies. It accounts for less than 2% of all thyroid cancers, but results in nearly one-third of thyroid cancer–related deaths [1]. The median survival rate of patients with ATC is less than six months, with 90% of patients presenting with unresectable ATC at the time of diagnosis and most patients developing recurrent ATC even after complete tumor resection [2, 3]. Currently, there is no standard or effective therapy for ATC, and patient survival has not improved in over six decades [2, 4]. Thus, there is a pressing need to identify agent(s) that inhibits disease progression or metastasis, and improve the survival of patients with ATC.

Drug discovery and development are costly and time-consuming, and rarely focused on rare malignancies. This is partly because it takes an average of 15 years and costs approximately $800 million to bring a single drug to market; this cost would not be recouped for rare cancer indications. Thus, quantitative high-throughput screening (qHTS) of existing drugs has become an emerging strategy for identifying new drugs for rare diseases/cancers such as ATC [5, 6]. There are several advantages to using this strategy, including knowledge of the drug's pharmacokinetics, pharmacodynamics, and side effects. These data can lead to a more streamlined translation of any promising preclinical findings into Phase II and/or III clinical trials to test the efficacy of the compound. Another advantage of this approach is the ability to determine whether the compound(s) identified from qHTS could effectively target known molecules or pathways that are specifically altered in a given cancer type. For example, in ATC, *TP53*-inactivating mutations are common (42–55%), followed by mutations in genes involved in the PI3K/AKT/mTOR, EGFR/RAS/BRAF/MEK/ERK, and WNT signaling pathways [7]. Mutually exclusive activation mutations in *RAS* and *BRAF* occur in approximately one-fourth of ATC cases [7, 8]. Mutations in *PIK3CA* and *PTEN* occur in 17–23% and 12% of ATC cases, respectively [7, 9]. Thus, agents identified by qHTS could be tested to evaluate their effects on these known activated pathways.

In this study, we performed qHTS in multiple ATC cell lines and identified 100 active compounds that were enriched for inhibitors of epidermal growth factor receptor (EGFR) signaling and histone deacetylase (HDAC). One of the most potent compounds identified was CUDC-101, a first-in-class dual inhibitor of EGFR, HER2 and HDACs [10, 11]. We then confirmed its effective inhibition of HDAC and EGFR/RAS/BRAF/MEK/ERK in ATC cell lines, and demonstrated that CUDC-101 inhibited ATC cell proliferation, disrupted cell cycle progression, and induced caspase-dependent apoptosis. More importantly, CUDC-101 treatment inhibited ATC tumor growth and metastasis *in vivo*, resulting in prolonged survival in a mouse model of metastatic ATC. Response to CUDC-101 treatment *in vivo* was associated with increased histone H3 acetylation and decreased survivin nuclear staining in tumor tissues.

## RESULTS

### Quantitative high-throughput screening of drug library

**Figure 1:**
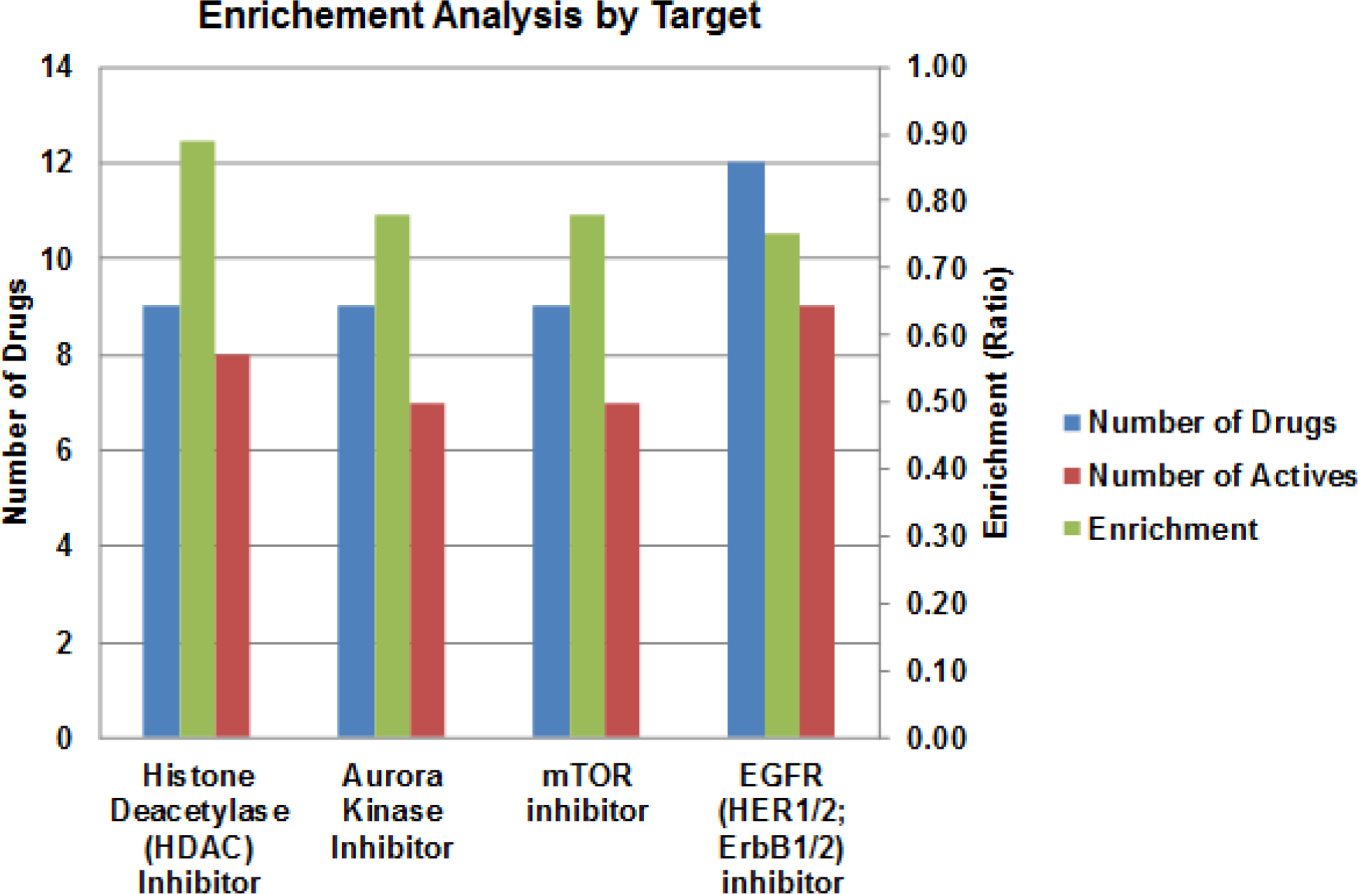
Results of qHTS in ATC cell lines Enrichment analysis was performed. The enrichment ratio (green, right Y-axis) represents the number of active drugs (red) vs. total drugs (blue, left Y-axis) by drug mode of action. An enrichment score of 0.7 was used as a cutoff.

Molecular heterogeneity among and within tumors is one of the major reasons that the efficacy of anticancer drugs is restricted to only a small subset of patients. To search for new therapies that are effective for broad groups of patients, we performed the drug library screening in three different ATC cell lines with distinct genetic background, 8505c, C-643 and SW-1736. Since *TP53* and the genes involved in PI3K/AKT/mTOR and MAPK pathways are frequently mutated in ATC, we first examined the mutation status of genes involved in these pathways. As summarized in Table 1, all three cell lines displayed *TP53*-inactivating mutations, as well as mutations in *EGFR*, *MET, BRAF, RAS, PI3K, PTEN*, and *mTOR*, suggesting that these cell lines are representative of human ATC. Thus, we used these three cell lines for the qHTS of the 3,282-compound library, and identified 100 compounds that were pan-active in the three ATC cell lines (Supplementary Table S1). To identify the top-ranking drug targets active against ATC cells, we performed enrichment analysis by mode-of-drug action on target classes that have at least five drugs in each class, and demonstrated that inhibitors of HDAC, aurora kinase, mTOR, and EGFR were the most active drug categories (Figure 1).

**Table 1:** Mutated genes involved in MAPK and PI3K pathways

Gene	8505c	C-643	SW-1736
*BRAF*	V600E		V600E
*EGFR*	R468K	R468K	
*HRAS*		G13R	
*MET*			E168D
*mTOR*		H419R	
*PIK3CB*			FRAME_SHIFT-50
*PIK3CG*			N522S
*PIK3R1*	M26I		
*PIK3R2*	S313P	S313P	S313P
*PTEN*		F341L	
*TP53*	R116G	R116Q, H178P, P72R	P72R, Q192*

### qHTS identifies CUDC-101, a dual EGFR, HER2 and HDAC inhibitor

**Figure 2:**
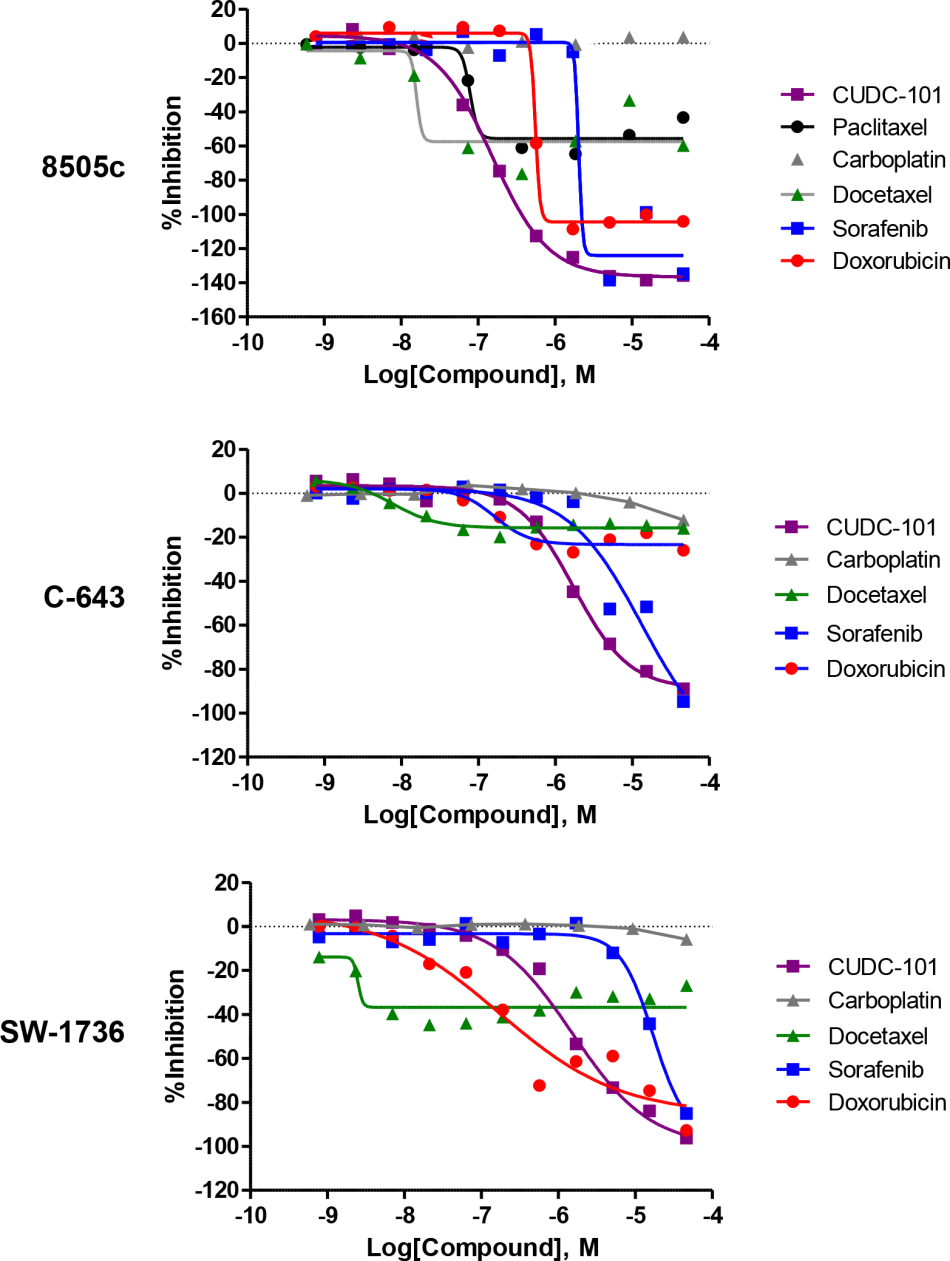
Comparison of dose-response curves for CUDC-101, paclitaxel, carboplatin, docetaxel, sorafenib, and doxorubicin Y-axis, percent inhibition; X-axis, drug concentration.

One of the highly active agents identified was CUDC-101, a first-in-class dual EGFR, HER2 and HDAC inhibitor [11]. CUDC-101 was the most active agent in all three ATC cell lines screened for inhibitors of EGFR and HDACs, with half-maximal inhibitory concentration (IC_50_) at 0.15 μM for 8505c, and 1.66 μM for both C-643 and SW-1736 cells. Current and past chemotherapy agents for ATC include sorafenib, paclitaxel, carboplatin, docetaxel, and doxorubicin as monotherapy or in combination with other agents, or as radiosensitizers to external beam radiation therapy [12–14]. Therefore, we compared the activity of CUDC-101 in the three ATC cell lines to these agents. As shown in Figure 2, CUDC-101 had better activity (maximum response and lower IC_50_) than sorafenib, paclitaxel, carboplatin, docetaxel, and doxorubicin.

### CUDC-101 inhibits cellular proliferation and cell cycle progression, and induces apoptosis in ATC cells

**Figure 3:**
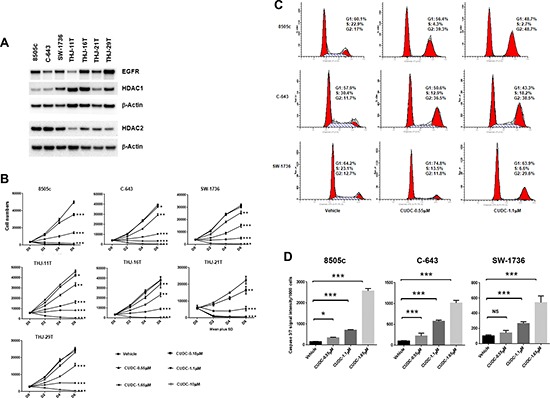
CUDC-101 inhibits ATC cell proliferation, and induces cell cycle arrest and apoptosis **(A)** Basal expression of HDAC1, HDAC2 and EGFR in ATC cell lines. **(B)** Cell proliferation assay. Error bars are mean ± SD. **(C)** Cell cycle analysis after 24 hours of treatment. **(D)** The Caspase-Glo 3/7 assay after 48 hours of treatment with CUDC-101. *p < 0.05, **p < 0.01, ***p < 0.001. NS, no significant difference.

As previously reported, HDAC1, HDAC2, and EGFR are over-expressed in ATC [15–19]. Therefore, we validated the antiproliferative activity of CUDC-101 using seven different ATC cell lines: 8505c, C-643, SW-1736, THJ-11T (*KRAS* mutation), THJ-16T (*TP53*, *RB*, *PI3KCA* mutations), THJ-21T (*TP53*, *RB*, *BRAF* mutations), and THJ-29T (*RB* mutation) [20]. We first determined the baseline expression of EGFR, HDAC1 and HDAC2 in these cell lines. As shown in Figure 3A, all the ATC cell lines expressed EGFR, HDAC1 and HDAC2, the targets of CUDC-101, under regular culture conditions. We then validated the activity of CUDC-101 on cell proliferation, and found dose- and time-dependent inhibition of cellular growth with cell death at higher concentrations of CUDC-101 in all seven ATC cell lines (Figure 3B).

To understand the mechanism by which CUDC-101 inhibited cellular proliferation and caused cell death at higher concentrations, we next assessed the effect of CUDC-101 on cell cycle progression and apoptosis using the three representative ATC cell lines used in the qHTS. Cell cycle analysis revealed that CUDC-101 treatment decreased the number of cells in the S phase and induced accumulation of cells in G2/M phase, which were dose-dependent (Figure 3C). To determine whether CUDC-101 induced caspase-dependent apoptosis, we performed caspase assay and found the drug induced an increase in caspase 3/7 activity (Figure 3D).

### CUDC-101 inhibits cancer cell migration and modulates epithelial-mesenchymal transition marker expression in ATC cells

**Figure 4:**
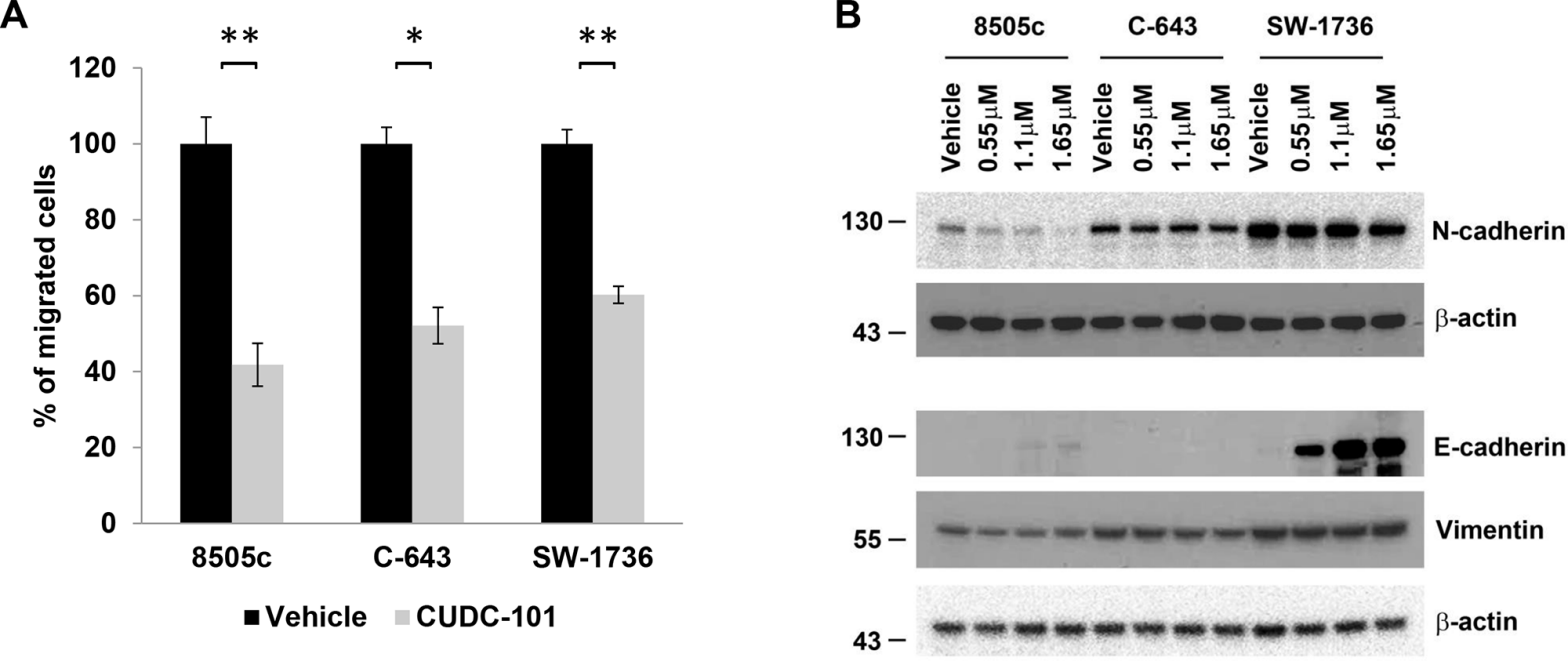
CUDC-101 inhibits ATC cell migration and regulates EMT marker expression **(A)** ATC cell migration with and without CUDC-101 treatment. The numbers of migrated cells were counted and calculated with the vehicle-treated cells as 100%. Data was for an average of three fields. Error bars are mean ± SD. *p < 0.05, **p < 0.01. **(B)** EMT marker expression. ATC cells were treated with CUDC-101 for 24 hours, and the blots were probed with the indicated antibodies to measure protein expression. Each corresponding anti-β-actin blot was used as loading control.

We next investigated whether CUDC-101 had any effect on cellular migration because ATC is a highly invasive cancer and the EGFR/RAS/BRAF/MEK/ERK pathway has been shown to regulate cellular migration and epithelial-mesenchymal transition (EMT) [21–23]. Compared to control, CUDC-101 significantly inhibited cellular migration in the ATC cell lines (Figure 4A). Given this effect on cellular migration, we evaluated whether CUDC-101 had any effect on EMT marker expression. ATC cells had basal expression of mesenchymal markers vimentin and N-cadherin (Figure 4B). In contrast, E-cadherin, a known tumorigenicity and tumor dissemination suppressor, was almost undetectable under the regular culture condition. CUDC-101 decreased N-cadherin level in 8505c and SW-1736 cells, but had minimal effect in the C-643 cell line (Figure 4B). For vimentin, CUDC-101 slightly reduced its level in 8505c and C-643 cells, but had no effect on its expression in SW-1736 cells. Interestingly, in SW-1736 cells, which had high basal expression of N-cadherin, treatment with CUDC-101 induced a significant increase in E-cadherin expression (Figure 4B), suggesting that this drug is effective even in cancer cells with high-levels of pro-EMT proteins.

### CUDC-101 inhibits HDAC and MAPK pathway, induces p21, and decreases survivin and XIAP expression in ATC cells

**Figure 5:**
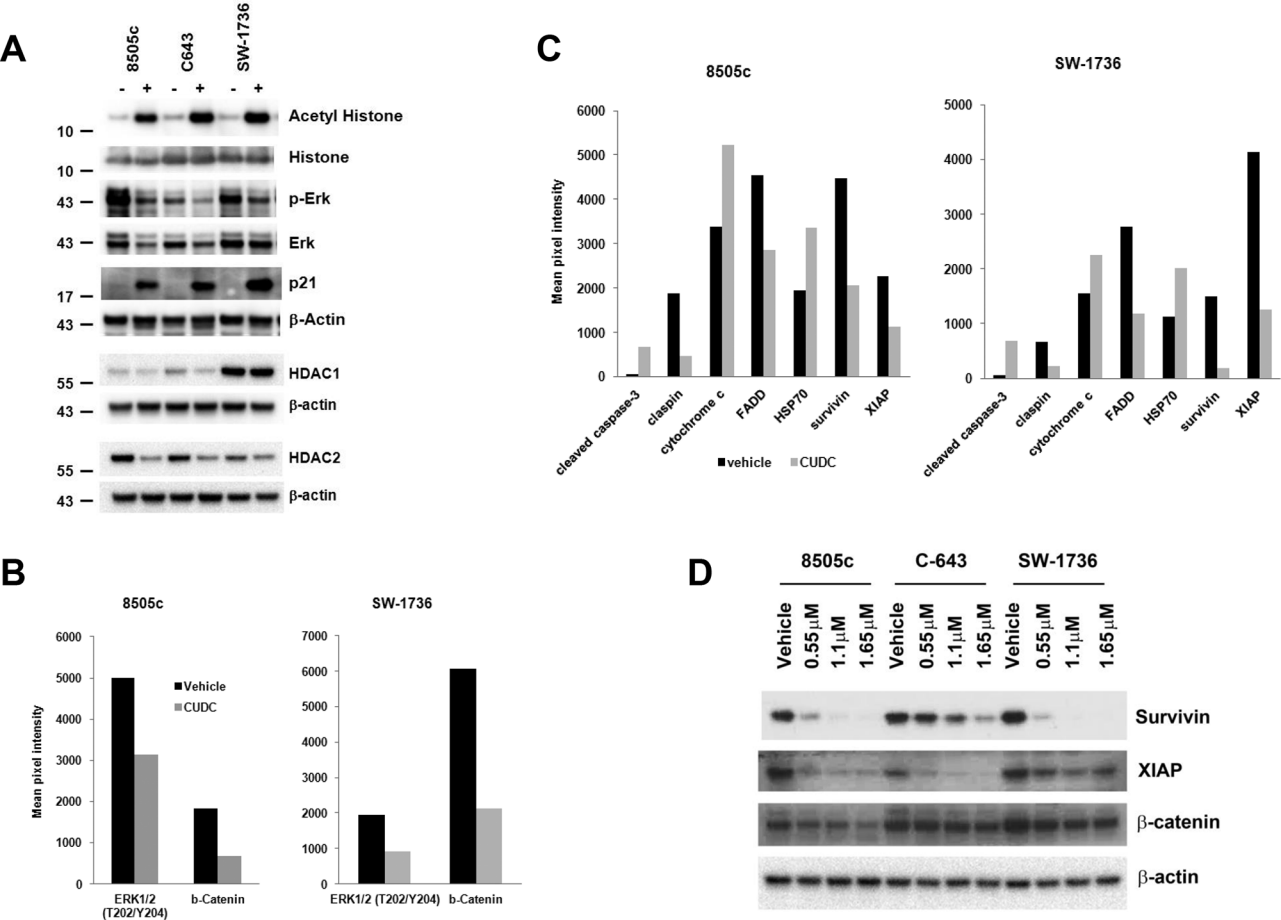
Protein targets of CUDC-101 in ATC cells **(A)** CUDC-101 inhibits HDACs and MAPK in ATC cells. **(B)** Targets of CUDC-101 identified by phospho-kinase array. Phospho-kinase arrays were performed using cell lysates treated with and without CUDC-101. Shown are those proteins that were altered with CUDC-101 treatment in both ATC cell lines with > 1.5-fold difference. **(C)** Differentially expressed apoptotic proteins with and without CUDC-101 treatment. Apoptosis arrays were performed. Shown are those proteins that were altered with CUDC-101 treatment in both ATC cell lines with > 1.5-fold difference. For A–C, ATC cells were treated with the vehicle or CUDC-101 at 1.1 μM for 24 hours. **(D)** CUDC-101 reduces the expression of survivin, XIAP, and β-catenin. Cells were treated with the vehicle or CUDC-101 for 24 hours.

CUDC-101 is thought to be a dual HDAC, EGFR, and HER2 inhibitor; therefore, we examined its inhibitory effect on HDAC and EGFR downstream pathways in ATC cells [24]. CUDC-101 effectively inhibited HDAC function with increased acetylation of histone, and reduced total ERK and phospho-ERK, downstream of EGFR signaling in all the three cell lines (Figure 5A).

As mentioned above, CUDC-101 inhibited cell cycle progression of ATC cells by reducing the cell numbers in the S phase and inducing arrest in the G2/M phase. Therefore, we examined the expression of p21, an important cell cycle regulator. Under regular culture condition, ATC cells had very low expression of p21, matching their aggressive cellular phenotype. CUDC-101 treatment induced an increase in p21 expression (Figure 5A). Furthermore, we also found that CUDC-101 not only inhibited HDAC function, but also reduced the expression of HDAC1 and HDAC2, especially in 8505c and C-643 cells (Figure 5A).

To determine if alternative activated pathways implicated in targeted therapy resistance are not activated with CUDC-101 therapy, and to further understand additional mechanism by which CUDC-101 may have an anticancer effect, we performed phospho-kinase protein arrays using 8505c and SW-1736 cells. As expected, CUDC-101 reduced the phosphorylation of ERK in both cell lines, but also decreased the expression of β-catenin (Figure 5B). We also performed apoptosis array to determine which molecules may mediate CUDC-101-induced ATC cell apoptosis. In both 8505c and SW-1736 cells, CUDC-101 treatment dramatically increased the level of cleaved caspase-3, which is consistent with the increased caspase 3/7 activity we observed (Figure 5C). More importantly, treatment with CUDC-101 also significantly reduced the expression of survivin and XIAP, known inhibitors of caspases [25] (Figure 5C). To verify the protein array results, we performed Western blots and confirmed CUDC-101 treatment reduced the level of survivin, XIAP, and β-catenin, which are upregulated in ATC and contribute to the highly aggressive behavior of this tumor (Figure 5D) [19, 26, 27].

### CUDC-101 inhibits tumor growth and metastases *in vivo*

**Figure 6:**
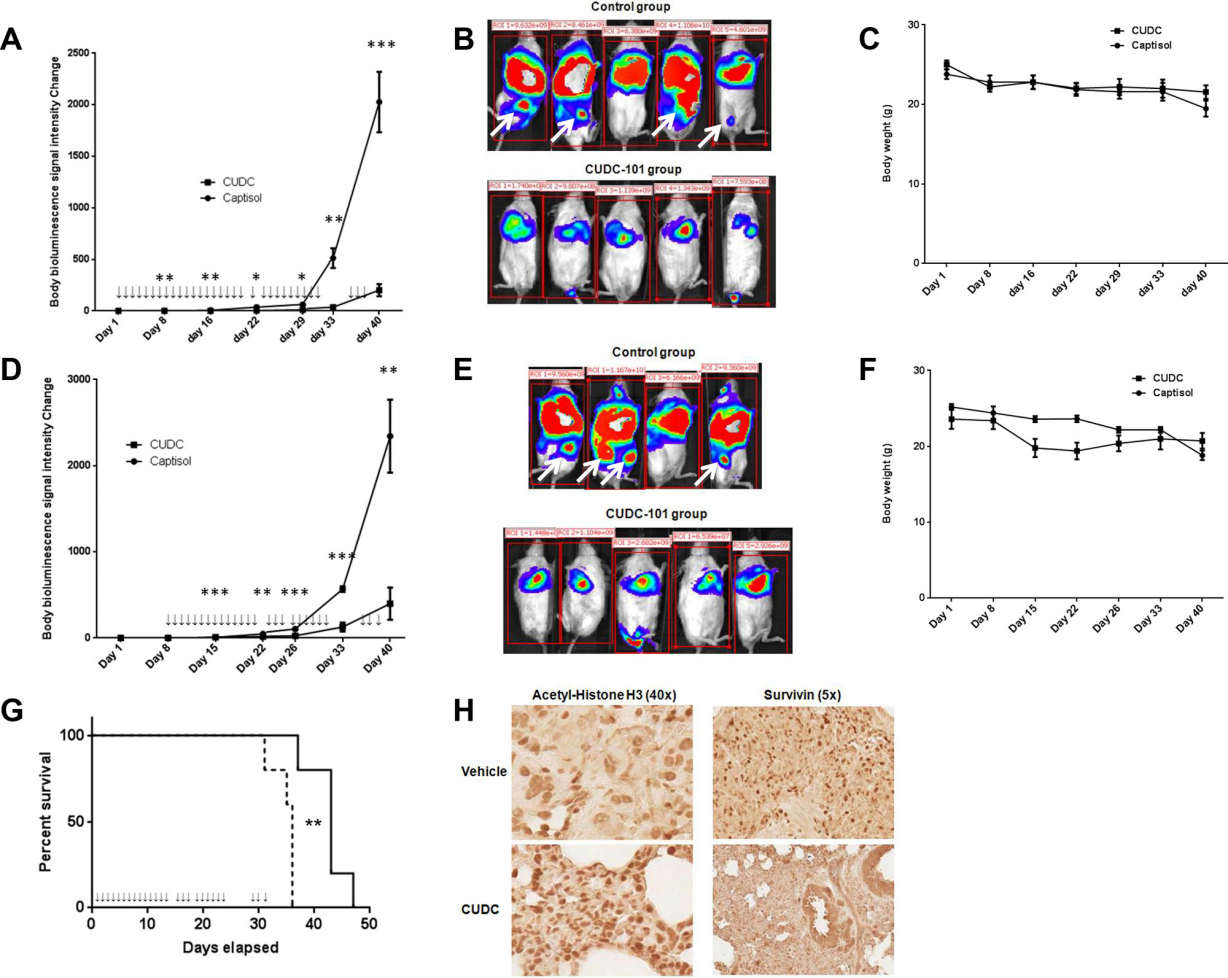
CUDC-101 treatment reduced ATC tumor growth and metastases *in vivo* **(A–C)** Mice (*n* = 5 for each group) were treated by intraperitoneal injection 20 minutes before tumor engraftment with 8505c-luc2 cells via the tail vein. (A) Luciferase activity fold changes. Treatment is indicated by the arrow (↓). Error bar is mean ± SEM. *p < 0.05, **p < 0.01, ***p < 0.001. (B) Whole-body images of treated and control mice at day 40 after cell engraftment. White arrows show sites of metastases to the liver and bone. (C) Mouse body weight changes. **(D–G)**, CUDC-101 inhibits tumor growth, metastases *in vivo,* and prolonged animal survival. Mice (*n* = 5 for each group) were engrafted with 8505c-luc cells via the tail vein and given CUDC-101 treatment 1 week after metastasis was established. (D) The Y-axis indicates the total change in luciferase activity. Error bars are mean ± SEM. The X-axis shows the time after engraftment, with the treatment indicated by the arrow (↓). *p < 0.05, **p < 0.01, ***p < 0.001. (E) Whole-body images of control and treated mice at day 40 of cell engraftment (one mouse in the control group died at this time). White arrows show sites of metastases to the liver and bone. (F) Mouse body weight changes with the treatment. (G) Survival of vehicle versus CUDC-101 treatment group. The first treatment day was indicated as day 1. *p* = 0.0034 by Cox log-rank test. H, Representative immunohistochemistry staining for acetyl-histone H3 and survivin. Five tumor samples per group (treatment and vehicle groups) were stained for acetyl-histone H3 and survivin.

To confirm our *in vitro* observations, the effect of CUDC-101 treatment was evaluated in a metastatic mouse model of ATC (8505c-Luc2) that recapitulates the aggressive nature of human ATC [28]. We first evaluated if pretreatment could reduce the rate of metastasis to assess the possible effects of CUDC-101 as an adjuvant therapy, and found that pre-treatment with CUDC-101 significantly reduced ATC metastasis in the mice (Figure 6A–6B). During treatment, we observed that mice treated with CUDC-101 developed gastrointestinal distress with diarrhea, but the symptoms were manageable with a gel meal and trans-gel food supplement, and with intraperitoneal fluid repletion using normal saline. The body weight of the treated mice was not significantly different from that of the control mice (Figure 6C). We then treated the mice with the established metastases, and found that CUDC-101 treatment significantly decreased tumor growth and metastases, and prolonged survival of treated mice (Figure 6D–6G). To determine biomarkers of response to CUDC-101 treatment, we examined metastatic tumor tissue from the *in vivo* experiments. We found increased levels of acetyl-histone H3 and decreased survivin nuclear staining in tumors that responded to therapy (Figure 6H). These results suggest that CUDC-101 may have potential as both adjuvant therapy and treatment for advanced ATC.

## DISCUSSION

Our study demonstrates an efficient and comprehensive approach to identify novel therapies for ATC. In this study, we performed qHTS using a large pharmaceutical collection with the goal of identifying new therapies that can be readily translated into the clinic. Multiple ATC cell lines with different genetic mutations were used in the screening. The qHTS identified 100 compounds that were active in all three ATC cell lines. Through enrichment analysis, the drug categories with the highest enrichment score were inhibitors of HDACs, aurora kinase, mTOR, and EGFR. CUDC-101, a first-in-class multi-targeted inhibitor of HDACs and receptor tyrosine kinases, was identified and confirmed to be a potent compound against ATC. *In vitro*, CUDC-101 treatment inhibited ATC cell proliferation and migration, caused cell cycle arrest, and induced caspase-dependent apoptosis. The anti-ATC activity of CUDC-101 occurred through the simultaneous inhibition of HDACs and the MAPK pathway downstream of EGFR, resulting in increased expression of p21 and E-cadherin and decreased expression of survivin, XIAP, N-cadherin, vimentin, and β-catenin. CUDC-101 exhibited antitumor effect *in vivo*, including the inhibition of tumor growth and distant bone metastasis, and prolonged survival in an ATC metastases mouse model.

To our knowledge, this is the first study to use the unique combination of a large clinical drug collection and multiple tumor cell lines to screen for novel therapies for ATC. This strategy offers several advantages. First, it offers a cost-effective approach to identify promising drugs for a rare and lethal malignancy such as ATC. Second, this approach presents a shortcut for bringing the laboratory-identified targets rapidly to clinical treatment. With the available information on clinical drugs, the multiple positive “hits” from the screen can be selected based on their pharmacokinetics and toxicity to achieve high efficacy with minimal toxicity. Third, using multiple cell lines with different genetic mutations involved in the targeted cancer assists in candidate selection. This selection is thus likely to be effective for a broad range of patient groups in the clinic since genetic heterogeneity among and within tumors is one reason that the efficacy of anticancer drugs is restricted.

Molecular studies indicate that ATC frequently harbor mutations in *TP53, BRAF, RAS, β-catenin, PIK3CA,* and *PTEN* [7, 27, 29–33]. Gene copy number and expression variations are also common in ATCs. Copy number gains have been reported for the following genes: *EGFR, VEGFR1* and *2, PDGFRA* and *B, PIK3CA*, *PIK3CB, KIT, PDK1, AKT1*, and *MET* [30]. Overexpression has been reported in EGFR, HDAC1 and 2, β-catenin, Aurora kinase, Cyclin E, Cyclin D1, PDGFRB, survivin, and HER-2 [15–19, 26, 34–36]. In contrast, the expression of E-cadherin is reduced in ATC [37]. It has also been observed that both ERK and AKT1 proteins are phosphorylated and, thus, activated in ATC [38, 39]. These studies emphasize that ATCs have a high degree of genetic abnormality that results in substantial expression variations in many genes and dysregulated signaling pathways such as EGFR/RAS/BRAF/MEK/ERK and PI3K/AKT [33]. CUDC-101 effectively inhibits these pathways in human ATC cells and targets multiple genes involved in cellular proliferation and migration making it an attractive anticancer agent.

Based on an improved understanding of the genetic basis of ATC, several targeted preclinical and clinical studies have been conducted to test new therapies. It has been reported that gefitinib, an EGFR inhibitor, reduces cellular proliferation and induces apoptosis in ATC cells, and slows tumor growth in a nude mouse model [18]. A Phase 2 trial of Pazopanib, a potent multi-targeted receptor tyrosine kinase inhibitor, was performed in patients with ATC. Although transient disease regression was observed in several patients, there were no confirmed RECIST responses [40]. Efatutazone, an oral PPAR-γ agonist, has been tested in combination with paclitaxel in a Phase 1 trial in ATC. Out of 15 patients, only one patient had a partial response [41]. Late intervention with the BRAF inhibitor, PLX-4720, has also been observed to induce tumor regression in an orthotopic mouse model of ATC with *BRAF* mutant cells [42]. Recently, it has been reported that vemurafenib, a BRAF inhibitor, resulted in near complete tumor regression in a patient with ATC who had an activating *BRAF* mutation [43]. A Phase II study in patients with ATC reported that imatinib, a multiple kinase inhibitor, shows some antitumor effect in advanced ATC cases [44]. Dasatinib, a Src kinase inhibitor, has been reported to inhibit cell proliferation in some ATC cell lines *in vitro* [45]. Another multiple kinase inhibitor, sorafenib, also shows antitumor activity in some patients with ATC [46]. Fosbretabulin, a vascular-disrupting agent, has been tested with carboplatin/paclitaxel (CP) against ATC. However, there was no significant difference in progression-free survival between CP and CP/fosbretabulin groups [47]. So far, long-lasting antitumor effects are rare most likely because of the high degree of genetic heterogeneity of ATC. With the multiple altered genes and pathways, ATC tumors can negate a drug's effect or escape from treatment by bypassing the level/factor being inhibited by a specific inhibitor. Therefore, simultaneous inhibition of multiple tumor cell pathways may result in more effective therapies.

CUDC-101 is a multi-targeted inhibitor of HDACs, EGFR, and HER2. The drug displays potent antiproliferative and proapoptotic activities against cultured cancer cells *in vitro* and in xenograft tumors *in vivo* [48]. Cancer cells that have acquired resistance to single-target EGFR inhibitors remain sensitive to CUDC-101 [24]. Mechanistic studies show that CUDC-101 integrates HDAC and EGFR/HER2 pathway inhibition, blocks and inhibits MET- and AXL-mediated signaling, and reduces cancer cell migration [24, 48]. Recently, a Phase I study of CUDC-101 reported that CUDC-101 was tolerated and showed some preliminary evidence of antitumor activity. Pharmacodynamic analysis in skin biopsy samples suggested that CUDC-101 effectively inhibits HDAC activity at the 275-mg/m^2^ dose level [11].

In this study, we found that CUDC-101 was the most potent compound in HDAC and EGFR inhibitor drug categories, two of the four most potent drug categories against ATC. Our validation study confirmed that CUDC-101 had potent antiproliferative and proapoptotic activities in multiple ATC cell lines. Overexpression of HDACs has been reported in multiple types of cancers, including ATC. HDAC inhibitors upregulate E-cadherin, inhibit cancer cell proliferation, migration and invasion as well as induce apoptosis and cell cycle arrest. [15, 49, 50]. Thus, by simultaneous inhibition of HDAC and the EGFR downstream pathway, CUDC-101 may offer a potential treatment for overcoming tumor resistance in ATC therapy. Our results demonstrated that CUDC-101 is effective in cell lines with gene mutations in both the MAPK and PI3K/AKT pathways further support this. For the first time we show that CUDC-101 treatment also reduces the expression of survivin, XIAP, N-cadherin, vimentin, and β-catenin, and restored p21 and E-cadherin expression in ATC cells. These results are consistent with CUDC-101-induced cell cycle arrest, apoptosis, and reduced cellular migration, also explain the reduced tumor growth and metastasis observed *in vivo*. Lastly, we show that acetyl-histone H3 and survivin level in tumor tissue is associated with response to CUDC-101 therapy, suggesting that these markers could be used as biomarkers for response to therapy.

In conclusion, we performed qHTS for 3,282 clinically approved drugs and pharmacologically active small molecules using three different ATC cell lines. The qHTS identified 100 active agents; the top four most active drug categories against ATC were inhibitors of HDAC, aurora kinase, mTOR, and EGFR. CUDC-101, a multi-targeted inhibitor of HDACs and tyrosine kinase receptors, was the most potent compound in the HDAC and EGFR inhibitor categories. We found that CUDC-101 had potent antiproliferative and proapoptotic activities in multiple ATC cell lines containing different genetic mutations involved in ATC. In a metastatic ATC mouse model, CUDC-101 treatment inhibited tumor growth and metastasis, and prolonged survival. Our findings suggest that CUDC-101 is a promising therapeutic agent for the treatment of ATC.

## METHODS

### Cell lines

Human ATC cell line 8505c was purchased from the European Collection of Cell Cultures (Salisbury, United Kingdom); C-643 and SW-1736 were purchased from Cell Lines Service (GmbH, Eppelheim, Germany); THJ-11T, THJ-16T, THJ-21T, and THJ-29T have been described previously [20]. Cell lines were authenticated by short-tandem repeat profiling on October 14, 2012, and August 30, 2013.

### Targeted sequencing of genes

The targeted sequencing of genes involved in the PI3K/AKT/mTOR, EGFR/RAS/BRAF/MEK/ERK, and WNT signaling pathways was performed using the Ion Torrent TargetSeq platform for 409 known tumor suppressor and oncogenes at the National Cancer Institute (NCI) Frederick Laboratory of Molecular Technology (Frederick, MD).

### Quantitative high-throughput drug screening

Two pharmaceutical collections were used: The National Center for Advancing Translational Sciences (NCATS) Pharmaceutical Collection (NPC) and the Mechanism Interrogation PlatE (MIPE) collection. Additional information on NPC drug libraries can be found at http://tripod.nih.gov/npc/. qHTS was performed in three ATC cell lines, 8505c, C-643, and SW-1736, as previously described [6]. The final concentration of the compounds was 0.5 nM to 46 μM. Tetraoctylammonium bromide, a toxin, was used as positive control (100% inhibition) and DMSO as negative control (0% inhibition).

### Cell proliferation, apoptosis, cell cycle, and migration assays

Cell proliferation assay was performed using the CyQuant kit (Life technologies, Grand Island, NY). Apoptosis was determined using the Caspase-Glo 3/7 assay kit (Promega) and normalized to the cell number.

Cell cycle analysis was performed at 24 hour after drug treatment on a FACScan using CellQuest software (BD Biosciences, San Jose, CA). Cell cycle data was analyzed using Modfit software (Verity Software House, Inc., Topsham, ME).

Cellular migration was determined using the Transwell chamber assay (BD Biosciences). After 24 hours of incubation with CUDC-101 or vehicle, cells were trypsinized, and an equal number of live cells were plated in each of the transwell chambers. After 22 hours of incubation at 37°C, the migrated cells were fixed, stained with Diff-Quik (Dade Behring Newark, NJ) and photographed.

### Western blot and antibodies

Following antibodies were used: anti-p21 (1:500), anti-vimentin (1:5,000), anti-E-cadherin (1:250), anti-survivin (1:2,000), anti-phospho-Erk1/2 (1:1,000), and anti-Erk1/2 (1:1,000)were from Cell Signaling Technology (Boston, MA); anti-N-cadherin (1:4,000) was from EMD Millipore (Billerica, MA); anti-β-catenin (1:250) and anti-XIAP (1:1,000) were from R&D Systems (Minneapolis, MN); and anti-β-actin (1:3,000) was from Santa Cruz Biotechnology (Dallas, TX).

### Phospho-kinase and apoptosis protein arrays

Human phospho-kinase (Catalog # ARY003B) array (detecting site-specific phosphorylation of 43 kinases and 2 related total proteins, and apoptosis array (Catalog # ARY009 detecting the expression level of 35 apoptosis-related proteins) were purchased from R&D Systems (Minneapolis, MN). Signal intensity of each spot was quantified with Image J (Bethesda, MD) and averaged for each specific protein.

### Immunohistochemistry

Tissues were fixed in 10% formalin, embedded in paraffin, sections were incubated with primary antibody (rabbit anti-acetyl-histone H3, 1:600, Cell Signaling Technology, Boston, MA; or rabbit anti-survivin, 1:500, Novus Biologicals, NB500–201, Littleton, CO) at 4°C overnight, and immunostaining was performed using Vectastain ABC and DAB kits (Vector Laboratories, Inc., Burlingame, CA, USA).

### *In vivo* ATC metastasis mouse model

An *in vivo* ATC metastasis mouse model was used to assess the effect of CUDC-101 [28]. The Animal Care and Use Committee of NCI/NIH approved the animal study protocol. 8505c-Luc2 cells were injected into the tail vein of six-month-old NOD. Cg-*Prkdcscid Il2rg^tm1Wjl^*/SzJ mice. Bioluminescence imaging was used to assess tumor burden using the Xenogen *in vivo* imaging system (Caliper Life Sciences. Inc., Hopkinton, MA) [28].

CUDC-101 (Curis, Lexington, MA) was reconstituted according to pharmacy manual directions. Eight days after tumor cell injection, widespread lung metastasis was established. The mice were randomized into two groups and injected with either CUDC-101 (120 mg/kg) or vehicle (10% captisol) daily, alternating between intravenous and intraperitoneal injections. Treatment continued until the first mouse reached humane euthanasia criteria endpoints. Then the mice were housed under normal care conditions with no further treatment and monitored daily.

To assess the effects of CUDC-101 on metastasis and as an adjuvant therapy, a pre-treatment mouse study was also performed. In this group, six-month-old NOD.Cg-*Prkdc^scid^*Il2rg*^tm1Wjl^*/SzJ mice were first injected intraperitoneally with either CUDC-101 (120 mg/kg) or 10% captisol solution. Twenty minutes later, the mice were injected with 8505C-*Luc2* cells via tail vein. CUDC-101 (120 mg/kg) or 10% captisol solution was injected daily. Treatment was continued until the first mouse reached humane euthanasia criteria endpoints. Then the mice were imaged, and euthanized by CO_2_ inhalation.

### Statistical analyses

qHTS data analysis was performed as previously described [6]. For validation of cell proliferation assays and animal study, statistical analyses were performed using GraphPad Prism 5 software (GraphPad Software, La Jolla, CA).

## SUPPLEMENTARY TABLE


