# Supplementary material for: Dual inhibition of HDAC and EGFR signaling with CUDC-101 induces potent suppression of tumor growth and metastasis in anaplastic thyroid cancer
Source: Oncotarget. 2015 Apr 13;6(11):9073–85. doi: 10.18632/oncotarget.3268 (PMC4496203; doi:10.18632/oncotarget.3268)
Supplement: Supplementary file 1 [file oncotarget-06-9073-s001.pdf]

SUPPLEMENTARY TABLE

Supplementary Table S1: Active compounds identified by qHTS

Compound Name	8505c cell line			C–643 cell line			SW–1736 cell line		
	Curve Class	IC_{50} (μM)	Efficacy (%)	Curve Class	IC_{50} (μM)	Efficacy (%)	Curve Class	IC_{50} (μM)	Efficacy (%)
(–)–Gossypol	–1.1	4.176	–117	–1.1	3.317	–91	–2.1	16.626	–99
A–674563	–1.1	2.349	–156	–2.2	18.655	–77	–2.1	6.619	–112
AHPN	–1.1	0.526	–110	–2.1	23.485	–115	–2.1	16.626	–113
Alisertib	–1.2	3.722	–62	–2.2	16.626	–73	–2.2	6.619	–59
Alvespimycin	–1.1	0.083	–140	–2.1	10.490	–104	–1.1	2.093	–88
AMG–47a	–1.1	1.321	–128	–1.1	5.258	–99	–1.1	2.635	–108
AMG–51	–1.1	4.686	–135	–2.1	18.655	–114	–1.1	5.258	–108
AMG–Tie2–1	–1.1	0.590	–131	–1.2	5.258	–55	–1.2	3.722	–92
AR–42	–1.1	0.166	–132	–2.1	3.722	–100	–2.1	5.258	–124
AV–412	–1.1	5.258	–119	–1.1	6.619	–104	–1.1	4.686	–98
Axitinib	–1.2	1.177	–65	–1.1	6.619	–90	–2.2	16.626	–61
AZ–23	–2.1	5.899	–133	–2.2	14.818	–51	–2.2	14.818	–93
AZD–7762	–1.1	0.418	–116	–1.1	4.686	–101	–1.1	1.177	–109
BAG–956	–2.1	16.626	–151	–1.2	0.743	–77	–1.2	0.662	–69
Bardoxolone methyl	–1.1	0.833	–101	–1.1	1.177	–91	–1.1	1.177	–100
Belinostat	–1.1	1.663	–131	–2.1	16.626	–99	–2.1	20.931	–104
BEZ235	–1.2	9.350	–57	–1.2	0.662	–56	–1.2	0.094	–54
BKM–120	–1.1	0.662	–92	–1.2	1.866	–50	–1.2	1.049	–59
BMS–777607	–2.1	13.207	–134	–2.2	20.931	–65	–2.1	13.207	–104
Bortezomib	–1.1	0.013	–107	–1.1	0.004	–106	–1.1	0.017	–84
BX–795	–1.1	4.176	–131	–1.2	4.686	–68	–1.1	1.663	–77
BX–795	–1.1	5.258	–148	–2.2	8.333	–86	–1.1	2.635	–97
Cabozantinib	–1.1	7.427	–130	–2.2	18.655	–59	–2.1	11.770	–103
Canertinib	–2.1	16.626	–134	–2.1	20.931	–99	–2.1	18.655	–98
Cantharidin	–1.2	1.607	–54	–1.1	2.660	–87	–1.1	6.682	–93
Carfilzomib	–1.1	0.004	–121	–1.1	0.012	–92	–1.1	0.030	–89
CAY10581	–1.1	2.957	–115	–1.1	7.427	–92	–1.1	2.957	–98
CAY10626	–2.1	11.770	–124	–2.1	5.899	–128	–2.1	5.258	–141
Chelerythrine chloride	–1.1	3.722	–109	–2.1	16.626	–98	–1.1	2.957	–100
Cortivazol	–2.1	16.626	–114	–2.1	26.351	–118	–1.4	4.686	–69
CUDC–101	–1.1	0.148	–141	–1.1	1.663	–91	–1.1	1.663	–103
DA–3003–1	–1.1	2.957	–137	–1.1	2.635	–101	–1.1	2.093	–101

(*Continued*)

Compound Name	8505c cell line			C–643 cell line			SW–1736 cell line		
	Curve Class	IC_{50} (μM)	Efficacy (%)	Curve Class	IC_{50} (μM)	Efficacy (%)	Curve Class	IC_{50} (μM)	Efficacy (%)
Deacetyl cortivazol	–2.1	20.931	–118	–2.1	23.485	–94	–1.4	4.686	–71
Degrasyn	–1.1	2.349	–134	–1.1	2.635	–74	–1.1	2.349	–96
Deguelin	–1.1	0.105	–134	–1.1	0.042	–91	–1.1	2.349	–92
Dovitinib	–1.2	5.258	–72	–2.2	29.566	–77	–2.1	18.655	–88
ENMD–981693	–2.1	14.818	–130	–2.1	23.485	–100	–2.1	16.626	–115
Entinostat	–1.1	0.235	–124	–2.2	10.490	–69	–2.2	6.619	–84
Fluvastatin	–1.1	0.235	–108	–1.2	1.482	–51	–1.2	2.349	–69
Foretinib	–1.1	3.317	–127	–2.1	10.490	–100	–1.1	3.317	–97
GDC–0941	–1.2	1.321	–80	–2.2	6.619	–76	–1.2	2.635	–81
GDC–0980	–1.2	0.935	–71	–1.2	0.469	–65	–1.2	0.372	–82
GSK–269962A	–1.2	4.176	–79	–1.2	2.635	–55	–2.2	10.490	–72
GSK–461364A	–1.1	0.015	–76	–2.2	23.485	–61	–2.2	1.177	–88
IKK16	–2.1	18.655	–131	–2.1	20.931	–110	–2.1	18.655	–102
IMD–0354	–1.1	0.037	–128	–1.1	0.037	–97	–1.1	0.469	–96
IPFK2	–1.1	0.469	–105	–2.2	16.626	–50	–1.2	1.663	–51
ISOX	–1.1	0.935	–134	–2.1	11.770	–103	–2.1	16.626	–129
Ispinesib	–1.1	0.011	–124	–2.1	26.351	–99	–2.1	8.333	–130
Itraconazole	–1.2	3.317	–54	–1.2	3.317	–55	–1.2	2.349	–77
Ixazomib	–1.1	0.042	–108	–1.1	0.013	–93	–1.1	0.132	–108
KU 0060648	–2.1	5.899	–125	–2.2	5.258	–70	–2.1	14.818	–107
Lestaurtinib	–1.1	0.469	–120	–1.1	3.722	–126	–2.1	3.722	–137
Lovastatin	–1.2	0.750	–71	–2.2	8.412	–75	–2.1	10.590	–97
Midostaurin	–1.2	0.418	–59	–1.2	1.482	–52	–1.2	1.049	–52
Mitoxantrone	–2.2	5.081	–52	–2.1	14.959	–114	–2.1	3.349	–133
MK–1775	–1.1	1.049	–102	–2.2	16.626	–58	–2.2	4.686	–79
MK–2206	–2.1	7.427	–156	–2.1	16.626	–99	–2.1	16.626	–102
Mocetinostat	–1.1	0.209	–112	–1.2	3.317	–64	–1.1	2.349	–65
Navitoclax	–1.1	0.209	–125	–2.1	5.258	–131	–1.1	7.427	–101
Nilotinib	–1.1	3.317	–109	–2.2	18.655	–69	–2.1	20.931	–99
NU–7441	–2.1	7.427	–135	–2.1	18.655	–103	–2.1	20.931	–99
NVP231	–1.1	1.663	–105	–1.2	7.427	–57	–1.2	8.333	–73
NVP–AUY922	–1.1	0.011	–116	–2.2	14.818	–55	–1.2	0.005	–53
Obatoclax	–1.1	2.093	–129	–2.1	6.619	–104	–2.1	18.655	–103

(*Continued*)

Compound Name	8505c cell line			C–643 cell line			SW–1736 cell line		
	Curve Class	IC_{50} (μM)	Efficacy (%)	Curve Class	IC_{50} (μM)	Efficacy (%)	Curve Class	IC_{50} (μM)	Efficacy (%)
PAC–1	–2.1	11.770	–88	–2.2	14.818	–90	–2.2	20.931	–52
Panobinostat	–1.1	0.024	–125	–1.1	0.148	–78	–1.1	0.083	–73
PD–166285	–1.1	0.235	–121	–2.1	6.619	–110	–2.1	3.317	–128
PF–05212384	–2.2	5.258	–79	–1.2	0.235	–73	–1.1	0.059	–83
PF–477736	–1.1	3.317	–91	–2.2	18.655	–90	–2.1	18.655	–116
PI–103	–1.2	0.743	–71	–1.2	0.372	–56	–1.2	0.264	–68
Pirarubicin	–2.2	13.336	–54	–1.2	2.113	–58	–2.1	2.985	–118
Ponatinib	–1.1	3.722	–127	–2.1	16.626	–121	–1.1	8.333	–116
Pracinostat	–1.1	0.296	–122	–2.2	4.686	–85	–2.1	5.258	–103
Regorafenib	–1.1	2.093	–121	–1.3	2.635	–79	–2.1	18.655	–94
Romidepsin	–1.3	0.001	–56	–1.1	0.005	–102	–1.1	0.053	–121
Salinomycin	–1.1	0.053	–108	–1.1	0.019	–97	–1.1	2.635	–103
SB1518	–1.1	1.482	–116	–2.1	18.655	–114	–2.1	16.626	–131
SCH–79797	–1.1	0.935	–110	–2.1	20.931	–101	–2.1	23.485	–103
SGI–1776	–2.2	20.931	–86	–2.1	26.351	–100	–2.1	26.351	–109
Silmitasertib	–1.1	4.686	–126	–2.2	13.207	–73	–1.2	4.176	–81
Simvastatin	–1.1	1.262	–109	–2.2	8.412	–77	–2.2	12.623	–67
Sorafenib	–1.1	2.635	–127	–2.1	13.207	–123	–2.1	16.626	–88
TAE–684	–1.1	1.177	–115	–2.1	18.655	–103	–1.1	2.957	–101
Telatinib	–2.1	23.485	–132	–2.2	20.931	–58	–2.2	18.655	–73
Tivozanib	–1.1	6.619	–127	–2.2	18.655	–53	–2.2	16.626	–64
Topotecan	–1.1	0.026	–129	–1.2	0.132	–53	–1.2	0.526	–69
Torin–2	–1.1	0.166	–124	–2.1	0.833	–106	–1.1	0.105	–110
Tozasertib	–2.1	18.655	–121	–2.2	23.485	–88	–2.1	18.655	–102
Trametinib	–2.2	18.655	–65	–1.2	0.372	–56	–2.2	0.418	–60
TW–37	–1.2	5.899	–78	–1.2	7.427	–81	–2.2	8.333	–70
Vandetanib	–2.2	14.818	–72	–2.1	18.655	–87	–2.1	20.931	–87
Vargatef	–1.1	0.935	–130	–2.1	11.770	–124	–2.1	13.207	–124
Vemurafenib	–2.1	14.818	–87	–2.2	26.351	–55	–2.2	1.177	–88
Vincristine	–1.1	0.007	–108	–1.2	0.053	–31	–1.2	0.019	–55
Volasertib	–1.1	0.030	–72	–2.2	18.655	–92	–2.1	20.931	–120
Vorinostat	–1.1	0.679	–128	–2.2	9.595	–93	–2.2	10.765	–110
Withaferin A	–1.1	0.590	–134	–1.1	1.663	–91	–1.1	2.093	–109
WYE–354	–1.2	10.490	–54	–2.2	13.207	–76	–2.2	7.427	–78
YM155	–1.1	0.002	–116	–1.1	0.009	–99	–1.1	0.004	–93
